# Promoting Sustainable Prescribing—A Case Study Presenting Experiences From Two Swedish Drug and Therapeutics Committees

**DOI:** 10.1002/prp2.70279

**Published:** 2026-06-20

**Authors:** Björn Ericsson, Ulf Lindahl, Johanna Villén, Marmar Nekoro, Helena Ramström, Björn Wettermark

**Affiliations:** ^1^ Gävleborg Drug and Therapeutics Committee Gävle Sweden; ^2^ Västernorrland Drug and Therapeutics Committee Härnösand Sweden; ^3^ Department of Pharmacy, Faculty of Pharmacy Uppsala University Uppsala Sweden; ^4^ Swedish Medical Products Agency Uppsala Sweden; ^5^ Health and Medical Care Administration Stockholm Sweden

**Keywords:** Drug and Therapeutics Committee, environment, pharmaceutical pollution, prescribing, rational drug use, sustainability, Sweden

## Abstract

During the past two decades, it has become evident that pharmaceuticals is an emerging environmental problem. In this study, we present the experiences from two Swedish Drug & Therapeutics Committees (DTCs) on how they integrate environmental considerations into their formularies and continuous professional education activities. A cross sectional study was performed describing activities to promote sustainable prescribing conducted during two decades by the DTCs in the Swedish regions of Gävleborg and Västernorrland, along with trends in sales of diclofenac and fluoroquinolones between 2000 and 2024. The two DTCs integrate environmental considerations in their formularies and continuous professional education activities. Further acitivities include monitoring of prescribing, dialogue with water suppliers and pharmacies, as well as information and communication to the general public. The total utilization of diclofenac for systemic use peaked with 127 kg in Gävleborg and 137 kg in Västernorrland in 2011, after which the amounts declined. The topical formulations were mainly sold as over‐the‐counter and increased until 2016, when it started to decline. The amounts of ciprofloxacin and norfloxacin increased until 2012 when they started to decline. This case study from two Swedish regions illustrates how environmental considerations can be incorporated into the DTC's mission to promote rational and responsible medication use. We believe it may be valuable for different stakeholders to show potential ways in how to promote a more sustainable use of medicines.

## 1 Introduction

Pharmaceuticals play an essential role in reducing mortality and morbidity. However, if they are improperly prescribed or used, the negative consequences can outweigh their benefits. In 1985, the World Health Organization (WHO) launched the concept of rational use of medicines, advocating that “all patients should receive medication appropriate to their medical needs, in doses meeting their own individual requirements, for an adequate period of time and at the lowest costs to them and the community” [1]. Drug and Therapeutics Committees (DTCs) were suggested as a suitable model to promote rational use of medicines, and WHO proposed that they should “ensure that patients are provided with the best possible cost‐effective and quality of care through determining what medicines will be available, at what cost, and how they will be used” [2].

The first DTCs focused on rationalizing the supply of medi‐cines in the hospital setting. In Sweden, the first DTC was established at Karolinska University Hospital already in 1961, with the main task to decrease the use of medicines with poor documentation and/or low cost‐effectiveness [3]. Over the years, the DTCs have broadened their focus. In 2013, a review of 207 articles described the evolution of DTCs as a consequence of changes in healthcare systems and the role of the prescriber [4]. In Sweden, the typical DTC consists of physicians, pharmacists, and nurses, but the base from which members can be recruited has widened over time. The initial formularies have evolved from limited lists of medicines into complete systems of medication management policies intended to ensure safe, appropriate, and cost‐effective use of pharmaceuticals in the interface between hospitals, primary care, patients, and the general public [4, 5].

Most DTCs produce and promote evidence‐based formularies for rational prescribing. In Sweden, a national law was instigated in 1996 making it mandatory for each of the 21 regions to have at least one DTC with a population‐focused approach, thus supporting both hospital‐based specialists and general practitioners (GPs) with guidelines or formularies for rational prescribing, drug utilization analyses, and continuous professional education [6, 7, 8]. In addition, Sweden started early to introduce policies to combat antibiotic resistance [9, 10]. Already in the 1980s, several measures were introduced, which were further strengthened by the creation of Strama (the Swedish strategic programme against antibiotic resistance) in 1995 as a national initiative aimed at combating antibiotic resistance [10, 11]. Strama operates as a network of authorities and organizations at national and local level, conducting a range of activities, including regular monitoring of antibiotic use and resistance patterns along with providing feedback to influence antibiotic prescribing habits. Strama has also developed and updated national treatment guidelines together with the Swedish Medical Products Agency and the Public Health Agency of Sweden and set targets on the number of antibiotic prescriptions. Training materials are provided and educational visits to healthcare professionals are organized, often in collaboration with the DTCs. There is both an app and an e‐learning course about Strama available in English [11]. Strama's innovative way of benchmarking through open comparisons of antibiotic use in regions, hospitals and healthcare centers has proven successful: the total number of antibiotic prescriptions has decreased in Sweden from 560 antibiotic prescriptions per 1000 inhabitants per year in 1992, to 271 per 1000 in 2024, without any measurable negative consequences for public health [12].

During the past two decades, it has become evident that not only antibiotics but also other pharmaceuticals are an emerging environmental problem [13, 14]. Most pharmaceuticals end up in rivers, lakes and oceans and they also reach groundwater [15, 16]. This, in combination with certain pharmaceuticals being toxic, persistent and accumulating in tissues, while also altering the behavior of aquatic organisms, has led to pharmaceutical residues becoming an increasing problem [17, 18, 19]. Diclofenac serves as a striking example of environmental impact, not least on vultures [20]. These birds died and the populations suffered catastrophic declines after the birds consumed carcasses of livestock treated with diclofenac which caused kidney failure. Studies on diclofenac have also shown negative effects on fish, including damage to the liver, kidneys, and gills [21]. In addition to diclofenac, ciprofloxacin, estradiol and ethinylestradiol are today classified as river basin‐specific pollutants (RBSPs) [22]. Diclofenac levels have been found to exceed environmental thresholds in several Swedish watercourses, posing risks to aquatic ecosystems. Furthermore, the use of antibiotics and their release into the environment can contribute to antibiotic resistance [23, 24]. Many stakeholders are important actors in reducing the environmental impact from pharmaceuticals, including the pharmaceutical industry, regulators, healthcare services and pharmacies. Since many pharmaceuticals reach ecosystems as a result of their usage after being prescribed, patients and healthcare professionals (e.g., prescribers and DTCs) are important stakeholders in reducing pharmaceutical pollution. This aligns well with proposals to integrate the environment into the definition of rational use of drugs, thus stating that each patient should receive medications appropriate to their clinical needs, in doses that meet their own individual requirements for an adequate period of time, at the lowest cost to them and their community, *considering the interconnection between people, animals, plants, and their shared environment* [25]. Recently, a decision‐making flowchart has been proposed to integrate environmental considerations in decision making therapeutics [26]. Examples of measures than can be taken by healthcare professionals to reduce pharmaceutical pollution include promotion of non‐pharmacological interventions, selection of medicines that are less harmful for the environment, where possible, since many medicines lack environmental information, medicines optimization, patient involvment in the prescribing process and deprescribing [26, 27, 28]. Interventions targeting societal culture offer the greatest leverage in reducing pharmaceutical pollution, as they involve raising public awareness and improving our knowledge base on the health, socioeconomic, and environmental impacts of pharmaceutical pollution [29].

Historically, DTCs paid little attention to the environmental aspects of pharmaceuticals. Pioneering steps were taken in the early 2000 by the DTC in Region Stockholm by integrating environmental considerations into the decision criteria for selecting pharmaceuticals to include in the formulary [7, 30]. The primary selection criterion is benefit/risk, documented according to strict criteria for evidence based medicine. However, a pharmaceutical with lower environmental impact may be preferred, provided its efficacy, safety, and cost‐effectiveness are comparable to those of existing alternatives. Some years later, Region Stockholm launched the knowledge support “Pharmaceuticals and Environment” with environmental information on active pharmaceutical ingredients (APIs) for human use on the Swedish market [31]. The environmental risk refers to the use of a pharmaceutical; however, information regarding potential risks associated with its manufacturing is lacking. Through Region Stockholm, this is freely available on Janusinfo.se [32]. During the following years, an increasing number of Swedish DTCs have started to adopt different activities focusing on pharmaceuticals in the environment, even though there is a challenge with the lack of environmental information available for many APIs [31, 32, 33].

The aim of this study is to present the experiences of two Swedish DTCs integrating environmental consideration into their continuous professional education initiatives and systems for monitoring and feedback on prescribing patterns. We also describe how the utilization of two groups of pharmaceuticals specifically harmful for the environment has developed over time in these regions. We believe it may be valuable for a range of stakeholders to show potential ways forward in how to promote a more sustainable prescribing and use of medicines.

## 2 Methods

This was an observational study describing how the Swedish DTCs operate in the two regions of Gävleborg and Västernorrland to promote a more sustainable drug use. A thorough review of official reports and websites published between 1988 and 2024, originating from these two regions and relevant national agencies, was undertaken. Additional material, for example, communication briefs, presentations, media outreach and publications from these regions known to the authors were also included. Measures taken by the DTCs are presented along with time series of sales data of diclofenac and two fluoroquinolones (ciprofloxacin and norfloxacin). These pharmaceuticals were selected because the Swedish Agency for Marine and Water Management decided to, in addition to estradiol and ethinylestradiol, classify diclofenac and ciprofloxacin as river basin‐specific pollutants (RBSPs) [22].

### 2.1 Setting

**FIGURE 1:**
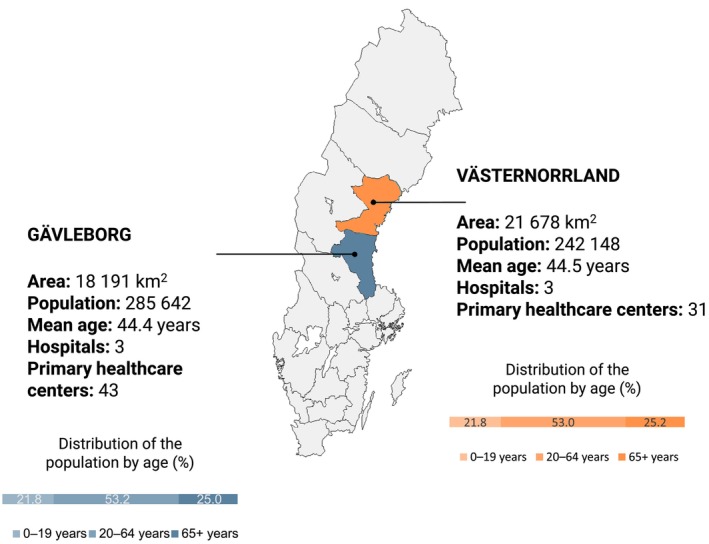
Characteristics of the population and healthcare systems in the Swedish regions of Gävleborg and Västernorrland.

In Sweden, the provision and financing of health services is a public sector responsibility, primarily resting with 21 regions. The two selected regions have populations of around 250 000 people each and cover large geographical areas with small cities and large sparsely populated rural areas (Figure 1).

The Swedish regions have the full budgetary responsibility for pharmaceuticals, both in hospitals and ambulatory care. However, they operate under the same national reimbursement system managed by the Swedish Dental and Pharmaceutical Benefits Agency, TLV [6]. The DTCs are acknowledged as the pharmaceutical policy experts in each region, and consequently act as advisors to politicians, civil servants, budget holders, and other stakeholders in all issues regarding pharmaceuticals. Further information about the two regions of Gävleborg and Västernorrland, including their structure for medicines management, can be found in Table S1.

### 2.2 Assessment and Presentation of DTC Activities

Measures taken by the two DTCs to promote a more sustainable prescribing of medicines are presented in the result section in chronological order. First, the general strategies and measures taken by each DTC are presented. This is followed by a more detailed description on how the DTCs have focused on diclofenac, as well as on two fluoroquinolones (ciprofloxacin and norfloxacin), a group of pharmaceuticals that pose an environmental risk. Diclofenac is not recommended by the DTCs. Before 2020, oral preparations of diclofenac were available over the counter (OTC), but they are now only available through prescription. Diclofenac for topical use is still available as OTC in Sweden. Ciprofloxacin is recommended by both DTCs. Further information about the selected medicines is found in Table 1.

**TABLE 1:** Environmental and clinical considerations for diclofenac and fluoroquinolones.

Aspect	Diclofenac	Fluoroquinolones
Environmental classification	Included in the EU Water Framework Directive Watch List [34]. Classified as a River Basin Specific Pollutant (RBSP) by Swedish authorities [22].	Ciprofloxacin classified as a River Basin Specific Pollutant (RBSP) by Swedish authorities [22].
Absorption and environmental risk	Topical skin absorption from dermal formulations is low around 6% compared to oral formulations. Risk being washed off (especially if the area applied is exposed to water shortly after application), thus may consequently constitute an environmental risk.	Risk of ecotoxicity and persistence in aquatic environments; contributes to antibacterial resistance [23, 24].
Indications for clinical use	Approved topically for mild to moderate local musculoskeletal pain (associated with muscular or joint injuries such as sport injuries), conditions are typically self‐healing. Approved orally for short term treatment of a wide range of mild to moderate acute pain conditions.	Historically used broadly for clinical infections (e.g., genitourinary, gastrointestinal, and respiratory tracts as well as infections of the bone), joints and skin; now more targeted, especially against gram‐negative bacteria. Ciprofloxacin has been considered essential for upper UTI (Urinary Tract Infection) with fever [35].
Health risks	Systemic use linked to increased cardiovascular risk [36].	According to EMA 2018 associated with risk of damage to connective tissue leading to increased risk for tendon ruptures and aortic aneurysms [37, 38].
Prescribing recommendations	Other interventions or analgesics with lower environmental impact are recommended [32].	Strama advises against use for lower UTI [11]. Ciprofloxacin is a first line treatment for upper UTI if accompanied with fever.
Market history for Sweden	Used since the early 1980s. Since 2020 oral diclofenac banned as OTC owing to the increased cardiovascular risks, available as prescription only.	Norfloxacin introduced 1986 for treatment of UTIs, withdrawn 2020. Ciprofloxacin introduced 1988, still in use.

### 2.3 Data Sources and Analyses of Sales Data

All data on medication sales were collected from the Swedish eHealth Agency. For diclofenac, prescription and OTC sales were separately presented for systemic use, for example, tablets/capsules (ATC; M01AB05) and for topical use (M02AA15). For fluoroquinolones, prescription sales were presented separately for two fluoroquinolones available on the Swedish market during the period: ciprofloxacin (J01MA02) and norfloxacin (J01MA06). The total amounts sold in kg of the selected medicinal products were determined using data on dispensed prescriptions and sold OTC‐medicines each year between 2000 and 2024. The mass (in kg) was based on the total mass of Defined Daily Doses dispensed for each substance [39]. For the topical formulation of diclofenac, which had no DDD, the amount was determined through multiplying the number of packages sold with the amount active substance in each package. No correction was made for whether the substance was in the form of a salt rather than pure API.

Trends in sales were presented descriptively as the amount of active substance in each region in kg/year. All data were analyzed with Microsoft Excel 2010. The study was conducted in accordance with the Basic & Clinical Pharmacology & Toxicology policy for experimental and clinical studies [40].

## 3 Results

### 3.1 General Strategies for the DTC Gävleborg

The DTC in Gävleborg started considering the environmental impact of medicines in 2002, following a political decision by the regional council. However, the decision did not specify how this should be implemented, nor were any additional resources allocated to support the work. Since then, this consideration has been further integrated into the DTCs' work with formularies and continuous professional education. These are briefly described below.

- *Environmental documentation in formularies*. Since 2019, the formulary presents environmental information for all included pharmaceuticals and those with the lowest environmental impact are selected for recommendation, when applicable. It is also presented if no environmental data is available. The main sources of environmental information are Janusinfo.se and Fass.se [32, 41]. The treatment recommendations also include non‐pharmacological advice. In some cases, pharmaceuticals have been assigned an “anti‐recommendation”. For diclofenac, such general advice has been in place since 2018.
- *Interventions targeted at prescribers*. Specific interventions have been directed at prescribers, with the ambition to reduce prescribing of medicines with a limited clinical value. Many of these medicines are also considered to have a problematic safety profile when used inappropriately. Examples of medicines include “potentially inappropriate medicines for the elderly”, antibiotics prescribed for viral infections, opioids, sedatives, and cough suppressants. Training for prescribers has been offered, both in the form of recurring information offered to each healthcare center four times per year, as well as lectures.
- *Monitoring of prescribing*. The DTC has systematically monitored prescribing patterns for all primary healthcare centers and hospital clinics. Data has been provided as feedback to prescribers and managers, as a form of benchmarking. For some therapeutic areas, prescribing quality indicators have been constructed and specific targets have been set from 2019 and onwards. At least four of these indicators (prescribing volumes of diclofenac, fluoroquinolones, sedatives, and benzodiazepines) have environmental implications. The target for diclofenac was set as 0 DDD in 2018. A prescription target for fluoroquinolones was established in 2003 and later revised in 2020, where it was set at < 5 DDD/1000 listed patients/day for primary healthcare centers. Strama has also separate targets for lower UTI, measuring fluoroquinolones as a share of total UTI‐antibiotic prescription [11].
- *Communication*. Through the DTC's own bulletin, “MIX”, articles concerning the environmental impact of pharmaceuticals, rational use of antibiotics and adherence to prescribing targets have been communicated in collaboration with the local Strama‐group to all healthcare professionals and managers in the region. On a few occasions, communication has also been directed to the general public through media about the environmental impact of pharmaceuticals.

### 3.2 General Strategies for the DTC Västernorrland

Since its formation in 1997, the DTC in Västernorrland has worked actively to reduce the environmental impact of pharmaceuticals. In its early years, general advice was issued to avoid unnecessary prescriptions and increase the use of start packages (smaller packages of medications) when initiating long‐term treatment with an ambition to reduce the environmental impact. Efforts also included educating the general public on the proper disposal of leftover medicines: encouraging returns to pharmacies rather than discarding them in the trash or flushing them down sinks or toilets. In 2021, a special task force focusing on “Pharmaceuticals and Environment” was formed, with 7 members representing different healthcare providers. Some of the measures taken are listed below.

- *Sustainability plan*. In Region Västernorrland's overall sustainability plan, it is specified that more patients should have their medications regularly reviewed in accordance with national requirements [42], that the number of antibiotic prescriptions should be reduced in line with Strama's objective to promote responsible use of antibiotics and that environmental requirements should be set and followed in the procurement of medicines, in accordance with the national criteria [43]. The region coordinates the procurement of medicines for the hospitals in its own region, but also three nearby regions.
- *Particularly harmful pharmaceuticals*. For particularly harmful pharmaceuticals, educational activities have been conducted, both through general internal training activities and targeted outreach to healthcare centers and hospital clinics. For diclofenac and ciprofloxacin, the DTC established specific prescribing targets aimed at reducing utilization. For diclofenac, the initial target was set in 2013 to max 22% of total NSAID DDDs in primary care. Gradually tightened to 4% by 2024–2025 through annual reductions. Fluoroquinolone use was limited to 0.96 DDD/1000 listed patients/day in 2005–2006. Since 2007 (still valid in 2025), no more than 10% of UTI antibiotic prescriptions for women in primary care should be fluoroquinolones (norfloxacin or ciprofloxacin). Each healthcare center has been provided with data showing their own prescribing compared to others, encouraging more informed prescribing practices. Several formulations are particularly problematic in terms of environmental risk, which the DTC takes into account in their recommendations, information and follow‐up. This applies to for example, medical patches and gels.
- *Reduced prescription volumes and waste management*. Various methods have been applied to reduce the total volumes of pharmaceuticals, including recommending physicians to initiate treatment with start packages, to generally recommend smaller package sizes in the event of short‐term complaints, to schedule reassessment or discontinuation of treatments when the benefits no longer outweigh the risks, and to refer patients to buy their medicines OTC when smaller amounts are needed to avoid unnecessary overprescribing. Medication reviews have also been applied for optimizing treatment for the elderly. The DTC monitors and sets targets for drug utilization reviews to be carried out for individual patients in the region. The DTC has also actively promoted non‐pharmacological methods and interventions in the recommendations. As a complement or replacement to medication treatment, the CBT (cognitive behavioral therapy) method is recommended, which can be used in the treatment of anxiety and depression. Visits to a physiotherapist can also supplement or replace medication for many conditions related to pain. The DTC formulary has a special chapter dealing with health behavior and prevention. Focus areas include avoiding tobacco, limiting alcohol, eating mindfully, and staying active every day—even a little movement makes a difference!
- *Dialogues with other organizations*. The DTC is in continuous contact with several other stakeholders, including the Swedish Water and Wastewater Association and local operators for water and sewage treatment plants, community pharmacies, and the payer organization for the healthcare system in the region. With all these actors, various joint projects have been carried out aiming for prudent and responsible use of pharmaceuticals, with the environmental aspect also taken into account.
- *Information and communication*. The DTC conducts continuous education and information activities. There are frequent meetings with most physicians and nurses in the region. Each healthcare provider in the region has assigned one of their employees to act as an environmental representative. All new environmental representatives receive special training about the environmental impact of pharmaceuticals and other environmental issues. A digital training course on the environmental impact of pharmaceuticals has also been developed.

### 3.3 Specific Actitivies Targeted at the Selected Pharmaceuticals

**FIGURE 2:**
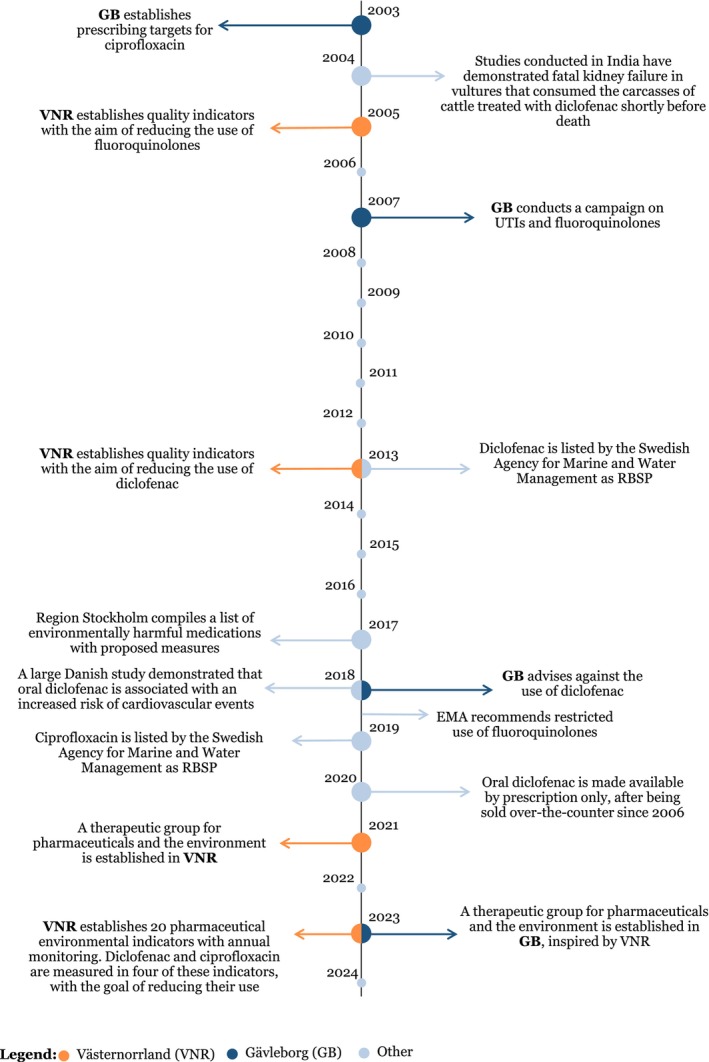
Timeline with examples of important activities targeted to reduce the environmental impact of diclofenac and fluoroquinolones.

Both DTCs early adopted activities to reduce the use of fluoroquinolones, with Gävleborg establishing prescribing targets in 2003 followed by Västernorrland in 2005 (Figure 2). The first measures to reduce diclofenac were taken by Västernorrland in 2013. Some key activities are presented in Figure 2.

### 3.4 Sales Data for the Selected Pharmaceuticals

#### 3.4.1 Diclofenac

**FIGURE 3:**
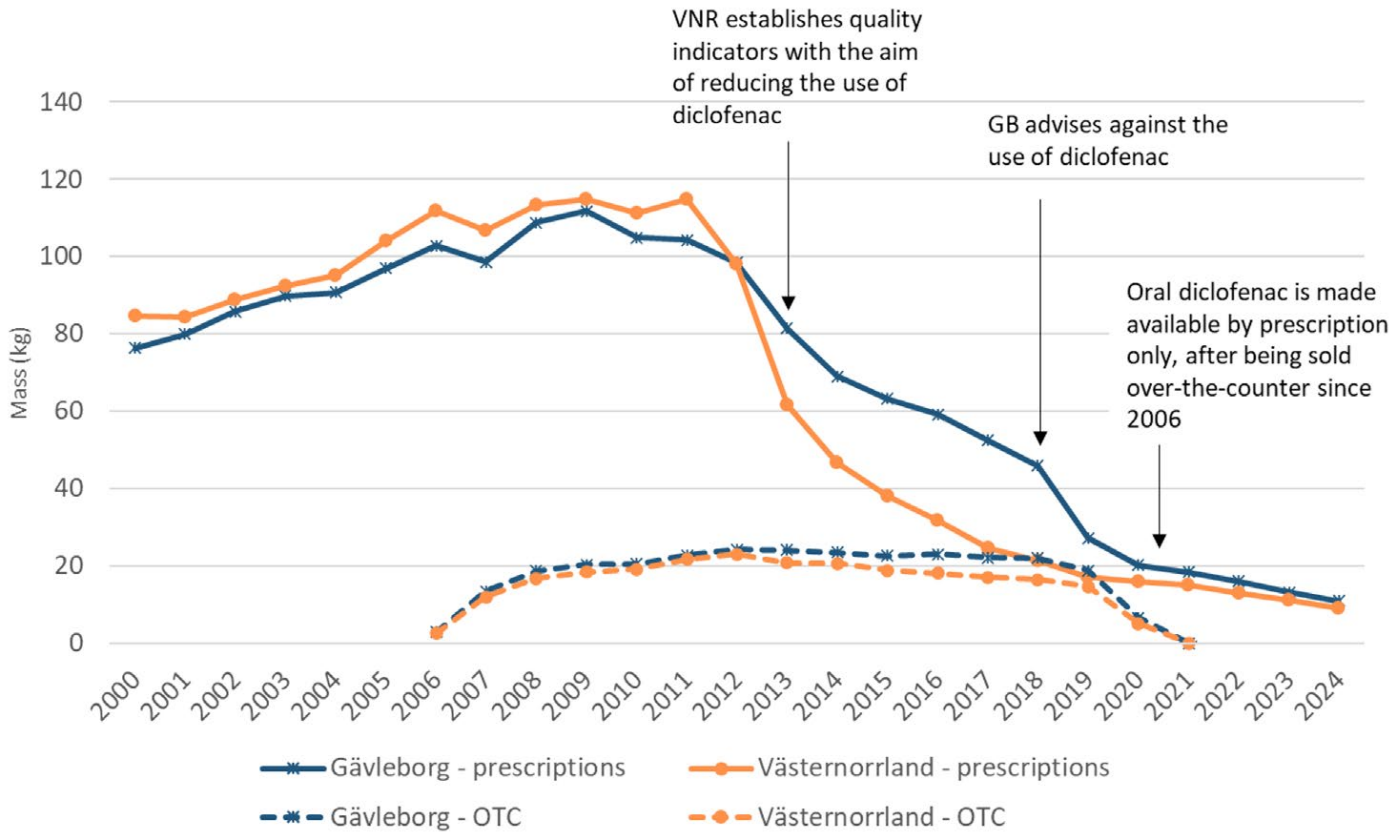
Sales of diclofenac for systemic use (kg) in Gävleborg (GB) and Västernorrland (VNR) 2000–2024.

The total amount of diclofenac for systemic use sold increased during the first 20 years and peaked with 127 kg in Gävleborg and 137 kg in Västernorrland in 2011, after which the amounts prescribed declined rapidly (Figure 3). The amounts sold as OTC remained at the same level with approximately 20 kg/year until 2020, when the Swedish Medical Products Agency classified the tablets as prescription only.

**FIGURE 4:**
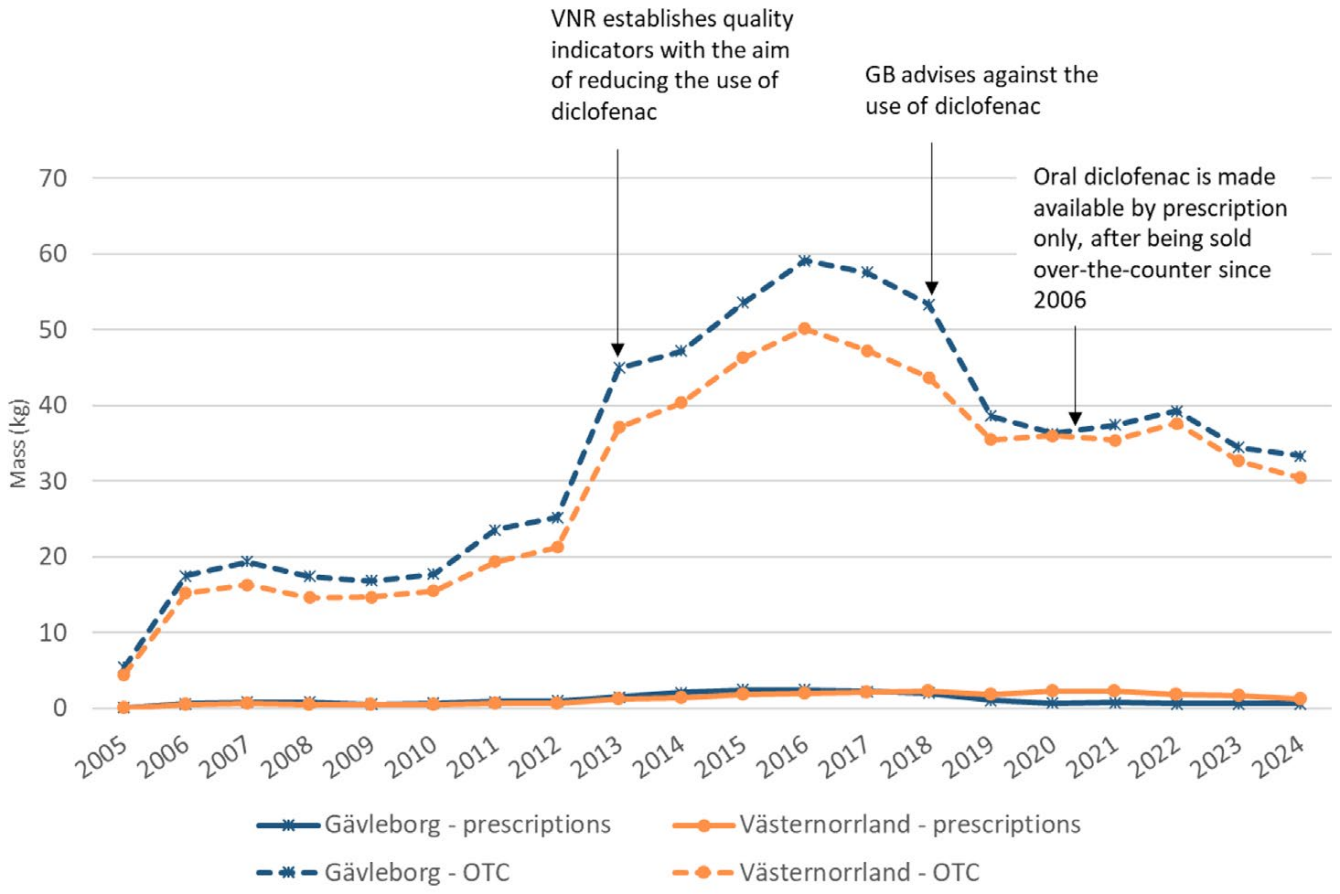
Sales of diclofenac topical formulations (kg) in Gävleborg (GB) and Västernorrland (VNR) 2005–2024.

The topical formulations became available in 2005 and the amount increased subsequently, particularly as OTC in both regions, until 2016 when the amounts started to decline (Figure 4).

#### 3.4.2 Fluoroquinolones (J01MA02 and J01MA06)

**FIGURE 5:**
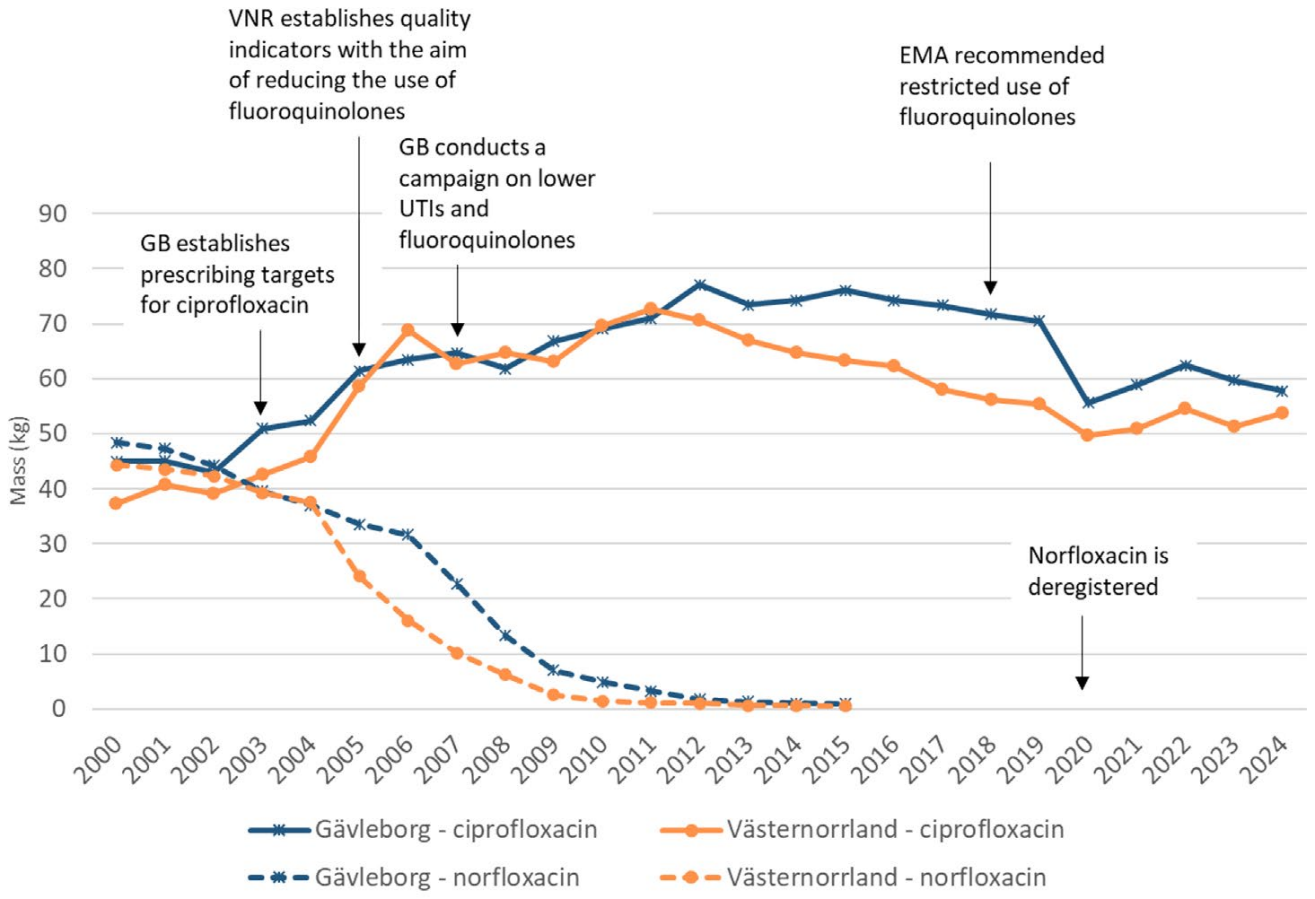
Sales of ciprofloxacin and norfloxacin (kg) in Gävleborg (GB) and Västernorrland (VNR) 2000–2024.

Between 37 and 50 kg each of ciprofloxacin and norfloxacin were sold in each region in 2000 (Figure 5). The amounts of norfloxacin declined until 2012, and the medicinal product was subsequently delisted from the market in 2020. The amounts of ciprofloxacin increased correspondingly until 2012 when the amount started to decline in both regions. The low amounts in 2020 can be explained by the pandemic.

## 4 Discussion

This case study of two Swedish regions, Gävleborg and Västernorrland, demonstrates how environmental considerations can be incorporated into the DTC's mission to encourage rational use of medicines. Environmental considerations are today integrated in formularies and continuous professional education activities. A plethora of further measures have been taken, with environmental considerations integrated throughout. These include monitoring of prescribing, requirements in tender processes, medication reviews, dialogue with other stakeholders as well as information and communication to the general public.

Sales trends of pharmaceuticals potentially harmful for the environment, specifically the active substance diclofenac and two fluoroquinolones (ciprofloxacin and norfloxacin), show a decline over time. Since other factors in addition to the work of the regions also play a role, such as the work of Strama, information from the EMA on the fluoroquinolones, and oral preparations of diclofenac becoming prescription‐only by the Swedish Medical Products Agency, corresponding trends are seen in other regions and nationally.

Overall, the breadth of activities conducted reflects a strong foundation and clearly builds on previous strategies applied by Swedish DTCs to influence prescribing [6, 7]. DTCs play an important role in improving quality of use of medicines including the environmental aspect, given their local ownership and large professional engagement. The Swedish DTCs facilitate a multi‐professional approach where physicians, nurses, pharmacists and administrators can meet to discuss pharmaceutical issues and policies from different perspectives, which further adds to their impact. Cross‐national comparisons of medicine prescribing are scarce, but some of those previously conducted show that Sweden has a comparatively high adherence to guidelines for rational choice of medicines compared to other European countries [44].

Reducing the use of diclofenac, which is not recommended by the DTCs, presents a clear synergy between environmental and patient health benefits. A large Danish study [36] demonstrated that diclofenac is associated with an increased risk of cardiovascular events, which ultimately led to the reclassification of oral diclofenac as a prescription‐only medication in Sweden in 2020. Our results indicate that this regulatory change did not lead to any marked increase in the sales of topical formulations of diclofenac in region Västernorrland or Gävleborg. In 2018, Region Gävleborg explicitly advised against the use of diclofenac, emphasizing both its cardiovascular risks and environmental impact. While it is difficult to isolate the direct effect of this recommendation, it seems to have contributed to a gradual shift in prescribing patterns. Still, there is a need for further efforts to reduce unnecessary diclofenac use. One potential long‐term solution is to incorporate environmental impact as a criterion for OTC availability [45]. However, such regulatory changes take time and additional measures are therefore needed, including public awareness campaigns and targeted initiatives at pharmacies beyond restricting the way how they are exposed [29, 46], to further limit diclofenac use and mitigate its environmental impact. A major challenge still lies in the pharmaceutical industry's promotional campaigns advocating the use of topical diclofenac.

While diclofenac has a negative impact on both cardiovascular health and the environment, it is not recommended by the DTCs and there are a number of pharmaceutical compounds that can replace diclofenac with lower environmental risk; fluoroquinolones are more challenging to substitute [32]. Even though fluoroquinolones pose both health and environmental risks, ciprofloxacin is still a recommended choice in certain situations. In order to mitigate negative effects, the approach should therefore be to further optimize the use. Previous studies have shown that ciprofloxacin can promote antibacterial resistance in concentrations well below therapeutic concentrations [23, 47]. EMA (PRAC, Pharmacovigilance Risk Assessment Committee) recommended restricted use of fluoroquinolones in relation to disabling and potentially long‐lasting side effects [37, 38]. However, since fluoroquinolones are prescription drugs only, the possibilities of control are in the hands of the authorities and healthcare providers. Formularies therefore need not only to list pharmaceuticals but also clearly recommend for which patient the drugs should be used. Strama has been proactive in guiding the use of fluoroquinolones, particularly ciprofloxacin, by developing precise treatment recommendations and advocating for the integration of targeted prescribing guidelines into formularies. Their initiatives aim to ensure that fluoroquinolones are prescribed judiciously, restricted to cases where they are genuinely necessary and suitable for specific patient groups, in alignment with evidence‐based protocols.

In some cases, switching to a pharmaceutical with a lower environmental risk may be possible; however, in most situations, a more holistic approach should be pursued. The mission of Sweden's regional DTCs, which promote rational pharmacotherapy by supporting evidence‐based prescribing and minimizing unnecessary or inappropriate use of medicines, is to recognize that appropriate use of medicines also benefits the environment. While Sweden's DTCs already work actively to promote evidence‐based prescribing, shared decision making, and systematic medication reviews, there is always room to further strengthen and formalize the integration of multi‐disciplinary evidence, particularly in relation to deprescribing and environmental sustainability [27, 28]. Although a new EU Urban Wastewater Directive has been adopted to address the treatment of pharmaceutical residues, upstream measures aimed at preventing pharmaceutical residues from entering wastewater in the first place will remain crucial. Furthermore, the Priority Substances Directive, which now has been approved by the European Parliament and includes diclofenac and several antibiotics (though not ciprofloxacin), sets ecological status thresholds to ensure environmental protection [34].

A general problem is the lack of environmental information of pharmaceuticals [31, 33, 48]. To facilitate informed decision‐making and to minimize the environmental impact of pharmaceuticals, stakeholders, including healthcare professionals and regulatory agencies, require easy access to reliable and up‐to‐date environmental information. The initiatives taken by Region Stockholm and the Swedish Pharmaceutical industry [31, 49, 50, 51] highlight the importance of web‐based knowledge support systems in providing environmental information on pharmaceuticals. Region Stockholm advocates that environmental information on pharmaceuticals should be provided by regulatory authorities, given their objectivity and access to both public and confidential data [31]. Access to correct and updated data is key for stakeholders including DTCs to promote sustainable practices and be able to balance human health needs with environmental protection through sustainable practices and rational use of medicines where environmental factors are included.

This study has several strengths. One key advantage is the use of comprehensive data on dispensed prescriptions and OTC use over a long time period, allowing for a robust analysis of trends in diclofenac, norfloxacin, and ciprofloxacin use. By converting sales data into kilograms, we provide a measure that is more relevant from an environmental perspective. However, we acknowledge that there are also some limitations to consider. The study is based on data from only two Swedish regions. Other regions have also implemented initiatives to reduce the environmental impact of pharmaceuticals, and their approaches may differ from those presented here. Sales of diclofenac, norfloxacin, and ciprofloxacin have decreased to a similar extent also in these regions. Another limitation is that online sales of OTC medicines, topical diclofenac, are currently not attributed to individual regions, except for those where the respective online vendors are officially registered. However, the national trend (including the online sales) appears to mirror the pattern presented in the article. In 2020 and 2021, all sales growth in the pharmacy market occurred online, with e‐commerce reaching nearly 20% of total market value and 28% of volume by 2022 [52]. Furthermore, while dispensed prescriptions and sales data offer important insights into trends, they do not necessarily reflect actual usage. A significant proportion of dispensed medications may never be used but instead remain stored in households or are disposed of improperly. This discrepancy between sold/dispensed and consumed medications is a common challenge in pharmacoepidemiological studies and highlights the need for complementary data sources, such as patient surveys or wastewater‐based analyses, to gain a more complete understanding of medication use and its environmental consequences [53].

In conclusion, this case study from two Swedish regions illustrates how DTCs can integrate environmental considerations into their mission, serving as an additional argument in promoting rational and evidence‐based use of medicines. Many of the initatives benefited from multidisciplinary collaboration with other actors including Strama, national authorities and the Swedish Water and wastewater association. We hope this could give inspiration for other countries on strategies to reduce the environmental burden of pharmaceuticals.

## Author Contributions

**Björn Ericsson:** conceptualization, formal analysis, writing – review and editing, data curation. **Helena Ramström:** methodology, validation, writing – review and editing. **Ulf Lindahl:** conceptualization, formal analysis, writing – review and editing, data curation. **Marmar Nekoro:** writing – review and editing, validation, methodology. **Johanna Villén:** methodology, visualization, formal analysis, writing – review and editing, validation. **Björn Wettermark:** conceptualization, writing – original draft, methodology, supervision, writing – review and editing, validation.

## Funding

This work was supported by the Uppsala Universitet.

## Ethics Statement

This study only presents aggregated publicly available data with no possibility to identify individual patients or healthcare providers. No ethical review board assessment was therefore conducted, in agreement with the Swedish legislation for conducting registry studies.

## Conflicts of Interest

B.E. and U.L. are members of the DTCs in the two regions. The authors declare no further conflict of interest.

## Supporting information


**Table S1:** The organization, support structures, and management of DTCs in the Swedish regions of Gävleborg and Västernorrland.

## Data Availability

Data available on request from the authors.
