# Supplementary material for: Promoting Sustainable Prescribing—A Case Study Presenting Experiences From Two Swedish Drug and Therapeutics Committees
Source: Pharmacol Res Perspect. 2026 Jun 20;14(4):e70279. doi: 10.1002/prp2.70279 (PMC13283205; doi:10.1002/prp2.70279)
Supplement: Supplementary file 1 — Table S1: The organization, support structures, and management of DTCs in the Swedish regions of Gävleborg and Västernorrland. [file PRP2-14-e70279-s001.docx]

***Supplementary material***

***Supplementary Table 1****. The organization, support structures, and management of DTCs in the Swedish regions of Gävleborg and Västernorrland.*

|  | ***Gävleborg*** | ***Västernorrland*** |
| --- | --- | --- |
| DTC organization and structure | The DTC Chairman is appointed by the Public Healthcare Services Committee, and members are appointed by the healthcare director.  The DTC has 16 members: physicians, nurses and pharmacists with broad representation across different types of healthcare and geography. The board has 4 meetings/year. | The DTC Chairman and members are appointed by the healthcare director.  The DTC has 10 members with broad representation across different types of healthcare and geography. The board has 6 meetings/year. |
| Formulary | Produced every second year, and includes medicines recommended as first and second-line choices for common diseases. | Produced annually, containing medicines recommended as first and second-line choices for common diseases. |
| Expert groups | 21 expert groups covering different therapeutic areas, including at least one pharmacist, one GP and one consultant doctor with the relevant speciality. In some cases, nurses are also represented. | 26 expert groups, with a similar structure and mission as Gävleborg. There is also a special expert group on pharmaceuticals and the environment. |
| Indicators and targets | The DTC defines “prescription targets”, which are revised yearly. Targets are categorized into economic, environmental and patient safety targets. Benchmarking statistics are presented for primary care units regularly, 2–12 times per year, depending on the target. | The DTC defines a number of quality indicators for rational use of medicines, and results are continuously monitored for each primary healthcare centre and hospital clinic. There are specific indicators addressing a more environmentally friendly medicine prescribing. |
| Support structure & activities | Both DTCs focus on:  – Producing the formulary  – Drug utilization analyses  – 2-day courses on pharmacology. Residents are obliged to pass this course in order to become specialists  – National collaboration on the managed introduction of new medicines  – Regular outreach visits to all healthcare centres | |
|  | – Information produced in a bulletin (MiX – 4 issues yearly) and on the web  – A yearly congress “Mellansvenskt Läkemedelsforum”, arranged together with 6 other DTCs located in the middle of Sweden  – Additional education on demand for clinics, local politicians and others  – DTC offer 4 yearly visits to each primary healthcare center  Activities are managed by 2–3 pharmacists employed by the Region. | – Information produced in a bulletin (4 issues/year), monthly newsletters for different target groups and on the web  – Drug utilization review for elderly patients  – Ensuring adequate supply of medicines  – Special projects on, e.g., local routines, optimizing electronic health records systems and ensuring appropriate management of medicines, when patients are discharged from hospitals  Activities are organized by an information group with of 8–9 persons (information physician, pharmacists and a nurse). |
| Cost responsibilities | In both regions: The budget for pharmaceuticals is decentralized to hospitals and primary healthcare centers. | |
|  | Primary healthcare centers have a medicine budget per listed patient, adjusted for age, disease burden (Adjusted Clinical Groups) and socioeconomic characteristics of patients. For hospitals in Gävleborg, strict cost responsibility applies to what is prescribed or procured. | Hospitals are responsible for the budget for specialist medicines, while primary care providers cover the cost of non-specialist medicines prescribed to individuals listed at each practice. |
